# Spiropyrimidinetriones: a Class of DNA Gyrase Inhibitors with Activity against Mycobacterium tuberculosis and without Cross-Resistance to Fluoroquinolones

**DOI:** 10.1128/aac.02192-21

**Published:** 2022-03-10

**Authors:** Gregory S. Basarab, Sandeep Ghorpade, Liezl Gibhard, Rudolf Mueller, Mathew Njoroge, Nashied Peton, Preshendren Govender, Lisa M. Massoudi, Gregory Thomas Robertson, Anne J. Lenaerts, Helena Ingrid Boshoff, Douglas Joerss, Tanya Parish, Thomas F. Durand-Reville, Manos Perros, Vinayak Singh, Kelly Chibale

**Affiliations:** a Drug Discovery and Development Centre (H3D), Division of Clinical Pharmacology, University of Cape Town, Observatory, Cape Town, South Africa; b Drug Discovery and Development Centre (H3D), Department of Chemistry, University of Cape Town, Rondebosch, Cape Town, South Africa; c Mycobacteria Research Laboratories, Department of Microbiology, Immunology and Pathology, Colorado State University, Fort Collins, Colorado, USA; d Tuberculosis Research Section, National Institute of Allergy and Infectious Diseases, Bethesda, Maryland, USA; e Infectious Disease Research Institute, Seattle, Washington, USA; f Center for Global Infectious Disease, Seattle Children’s Research Institute, Seattle, Washington, USA; g Entasis Therapeutics, Inc., Waltham, Massachusetts, USA; h South African Medical Research Council Drug Discovery and Development Research Unit, Institute of Infectious Disease and Molecular Medicine, University of Cape Town, Rondebosch, Cape Town, South Africa

**Keywords:** spiropyrimidinetrione, *Mycobacterium tuberculosis*, tuberculosis, DNA gyrase, drug resistance

## Abstract

Described here is a series of spiropyrimidinetrione (SPT) compounds with activity against Mycobacterium tuberculosis through inhibition of DNA gyrase. The SPT class operates via a novel mode of inhibition, which involves Mg^2+^-independent stabilization of the DNA cleavage complex with DNA gyrase and is thereby not cross-resistant with other DNA gyrase-inhibiting antibacterials, including fluoroquinolones. Compound 22 from the series was profiled broadly and showed *in vitro* cidality as well as intracellular activity against M. tuberculosis in macrophages. Evidence for the DNA gyrase mode of action was supported by inhibition of the target in a DNA supercoiling assay and elicitation of an SOS response seen in a *recA* reporter strain of M. tuberculosis. Pharmacokinetic properties of 22 supported evaluation of efficacy in an acute model of M. tuberculosis infection, where modest reduction in CFU numbers was seen. This work offers promise for deriving a novel drug class of tuberculosis agent without preexisting clinical resistance.

## INTRODUCTION

Tuberculosis (TB) remains an urgent worldwide health problem despite the nearly 140-year time lapse since the causative bacterium, Mycobacterium tuberculosis, was identified by the German physician Robert Koch and *in vitro* culture methods were developed (1, 2). The discovery of streptomycin and *para*-aminosalicylic acid for the treatment of TB in the 1940s offered hope for a cure; significantly, coadministration of the two drugs along with isoniazid proved superior to each alone, heralding the use of combination therapy. Through the decades, improved combination therapies have been developed to treat TB, including the current most used quadruple regimen of rifampin, isoniazid, pyrazinamide, and ethambutol (3). However, the best-case treatment regimens require 6 months of therapy and suffer substantially from problematic side effects and poor patient compliance. Furthermore, resistance to each of the individual first-line drugs has developed, as has multidrug-resistant (MDR) and extensively drug-resistant (XDR) strains of M. tuberculosis (4, 5). MDR-TB minimally shows resistance to isoniazid and rifampin and oftentimes includes resistance to moxifloxacin (6). Fluoroquinolones (FQs) such as moxifloxacin have been used clinically in combinations for both first- and second-line treatments; indeed, the combination of moxifloxacin with rifapentine, isoniazid, and pyrazinamide has recently been shown to reduce the treatment time for TB cure to 4 months (7). Hence, intense efforts to discover new compounds with novel modes of action and no cross-resistance to currently used drugs must continue for improved regimen development.

Spiropyrimidinetriones (SPTs) represent a new class of antibacterial agent that operate by inhibition of DNA gyrase in a manner differentiated from FQs such as moxifloxacin and other DNA gyrase-inhibiting antibacterials, including ATP-competitive (GyrB) inhibitors such as novobiocin and naphthyridones/aminopiperidines such as GSK000 (8–10). The antibacterial spectrum of the SPT class includes activity against Gram-positive and fastidious Gram-negative pathogens, with the most advanced member, zoliflodacin (previously AZD0914 and ETX0914), currently in phase 3 clinical trials for the treatment of gonorrhea caused by Neisseria gonorrhoeae (11, 12). It follows that since SPTs inhibit DNA gyrase, as does moxifloxacin, then activity against M. tuberculosis would be expected. Towards assessing this utility, Entasis Therapeutics, which is developing zoliflodacin in collaboration with the Global Antibiotic Research and Development Partnership (GARDP), provided a set of 24 SPT analogues for characterization against M. tuberculosis. The analogues were screened under various culture conditions against M. tuberculosis (13), and the attributes of the leading candidate, 22, from the screening effort are described here. In the present study, detailed are the microbiological characteristics, *in vitro* and *in vivo* pharmacology, and efficacy in a mouse model of acute TB infection for this lead candidate.

## RESULTS

### Anti-M. tuberculosis activity of spiropyrimidinetriones.

Zoliflodacin and the set of 24 analogues provided by Entasis (Tables 1 to 3) were evaluated for anti-M. tuberculosis activity using the H37Rv strain and reported as the MIC representing the lowest drug concentration inhibiting bacterial growth by 90%. Evaluations were carried out in five different Middlebrook 7H9-based growth media where various supplements were added either as carbon sources (dipalmitoylphosphatidylcholine [DPPC], glucose, or cholesterol), surfactants (Tween or tyloxapol), or additives (albumin-dextrose-catalase [ADC], bovine serum albumin [BSA], or Casitone) to mimic the *in vivo* environment (14–17). The data for moxifloxacin as a positive control were included in Table 3, as it is the DNA gyrase inhibitor most widely used for the treatment of TB clinically. The structural integrity and high purity (>95%) of all the compounds were confirmed by liquid chromatography-mass spectrometry (LC-MS) analysis, demonstrating that the 24 samples had not degraded despite being stored for multiple years as solid samples. The analogs can be stratified by the R-substituents on the benzisoxazole scaffold, with oxazolidinone (13 examples; Table 1) and N-linked triazole (6 examples; Table 2) substituents being most common. SAR of SPTs with oxazolidinone and triazole substituents have been presented in the literature in the context of activity against Gram-positive and Gram-negative pathogens (18, 19). Table 3 contains the data for miscellaneous other compounds, including 24, that has a difluorobenzene rather than a benzisoxazole scaffold while having the opposite absolute stereochemistry on the morpholine ring, which is known to be required for activity (20, 21). As expected, 24 did not show activity at the highest concentration tested in any of the M. tuberculosis MIC assays, thereby serving as a negative control. Notably, about half of the compounds with the oxazolidinone R-substituent were more active than zoliflodacin against M. tuberculosis H37Rv across the various medium conditions (Table 1). The most active compound 6, having a tetrahydrofuran fused to the oxazolidinone ring, was 4- to 12-fold more active than zoliflodacin. Compounds 2, 3, and 5 displayed better activity than zoliflodacin against M. tuberculosis; these were previously shown to have similar activity against other bacterial species, including against the zoliflodacin target pathogen N. gonorrhoeae (19). Except for 19, the compounds shown in Table 2 incorporating an N-linked triazole substituent on the benzisoxazole showed higher activity than zoliflodacin against M. tuberculosis. Compound 17 also showed higher activity than zoliflodacin against Gram-positive bacterial pathogens (Staphylococcus aureus, Streptococcus pneumoniae, and Streptococcus pyogenes), while compounds 14, 15, 16, and 18 showed similar or lesser activity (18). Compound 14 showed the highest anti-M. tuberculosis activity (MIC, 0.24 to 1.6 μM) of all 24 compounds assessed. However, the N-linked triazole compounds as a class showed genotoxicity in a mouse lymphoma assay (MLA) that detects micronucleation aberrations, thereby rejecting consideration for their development as anti-TB agents (11, 18). Finally, 22 (11) displayed considerably higher activity (averaging about 10-fold) than zoliflodacin against M. tuberculosis and showed the highest activity of compounds with an R-substituent other than oxazolidinone or triazole (Table 3).

**TABLE 1:**
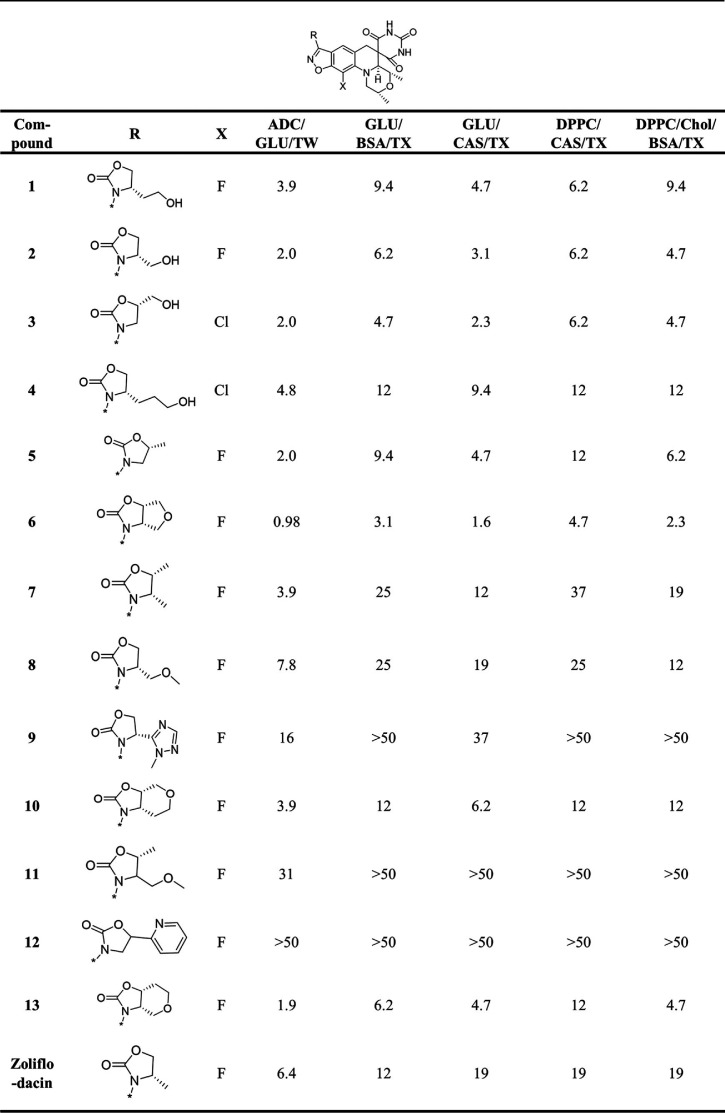
MIC (μM) for SPT oxazolidinones against M. tuberculosis H37Rv in 7H9 medium with various supplements^*a*^

a ADC, albumin-dextrose catalase; GLU, glucose; TW, Tween; CAS, Casitone; TX, tyloxapol; Chol, cholesterol.

**TABLE 2:**
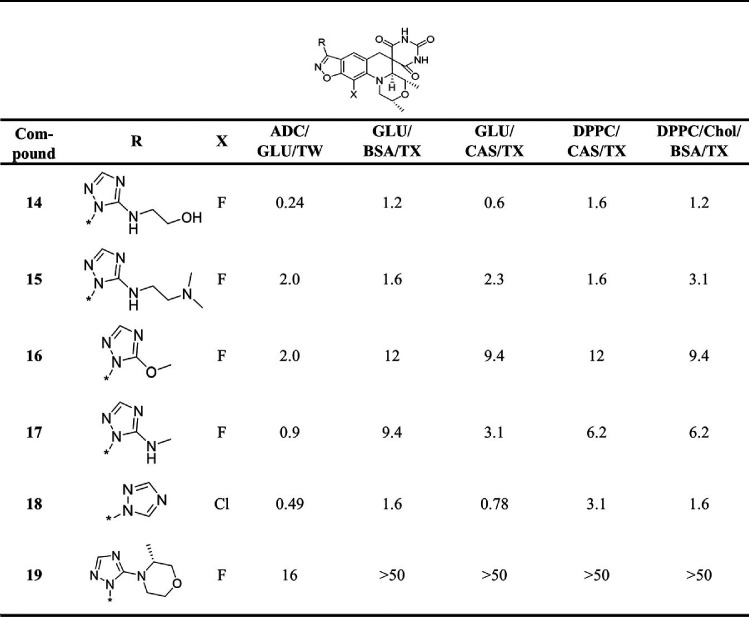
MIC (μM) for SPT N-linked triazoles against M. tuberculosis H37Rv in 7H9 media with various supplements^*a*^

a ADC, albumin-dextrose catalase; GLU, glucose; TW, Tween; CAS, Casitone; TX, tyloxapol; Chol, cholesterol.

**TABLE 3:**
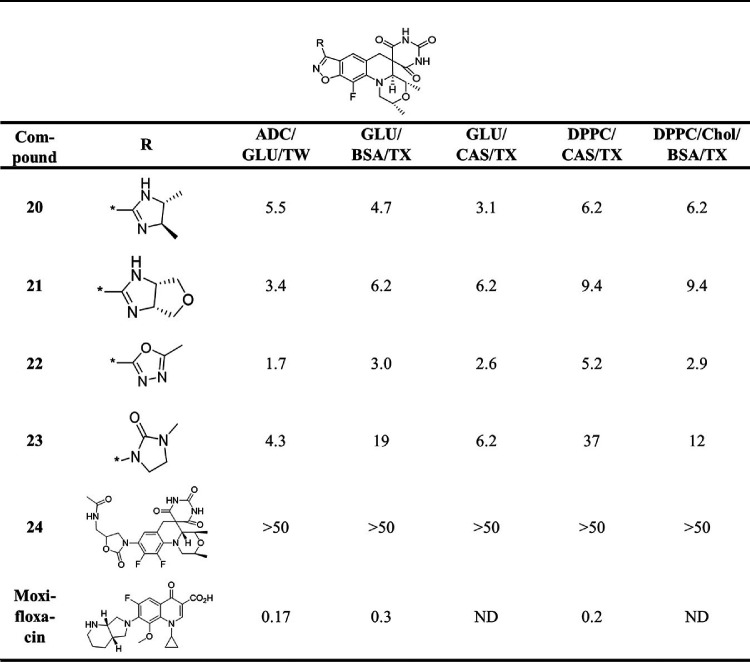
MIC (μM) for miscellaneous SPT compounds and moxifloxacin against M. tuberculosis H37Rv in 7H9 media with various supplements^*a*^

a GLU, glucose; TW, Tween; CAS, Casitone; TX, tyloxapol; Chol, cholesterol; ND, not determined.

Compounds that displayed lower and more consistent MICs across the medium conditions and do not have reported issues of genotoxicity were favored for further characterization, namely, 2 (MIC, 2.0 to 6.2 μM), 3 (MIC, 2.0 to 6.2 μM), 6 (MIC, 0.98 to 4.7 μM), 20 (MIC, 3.1 to 6.2 μM), and 22 (MIC, 1.7 to 5.2 μM). As 22 was specifically reported to show no genotoxicity at the highest concentration tested in the MLA (11), it was selected for further microbiological and pharmacokinetic (PK) profiling *en route* to assessing efficacy in a mouse model of TB infection.

**FIG 1:**
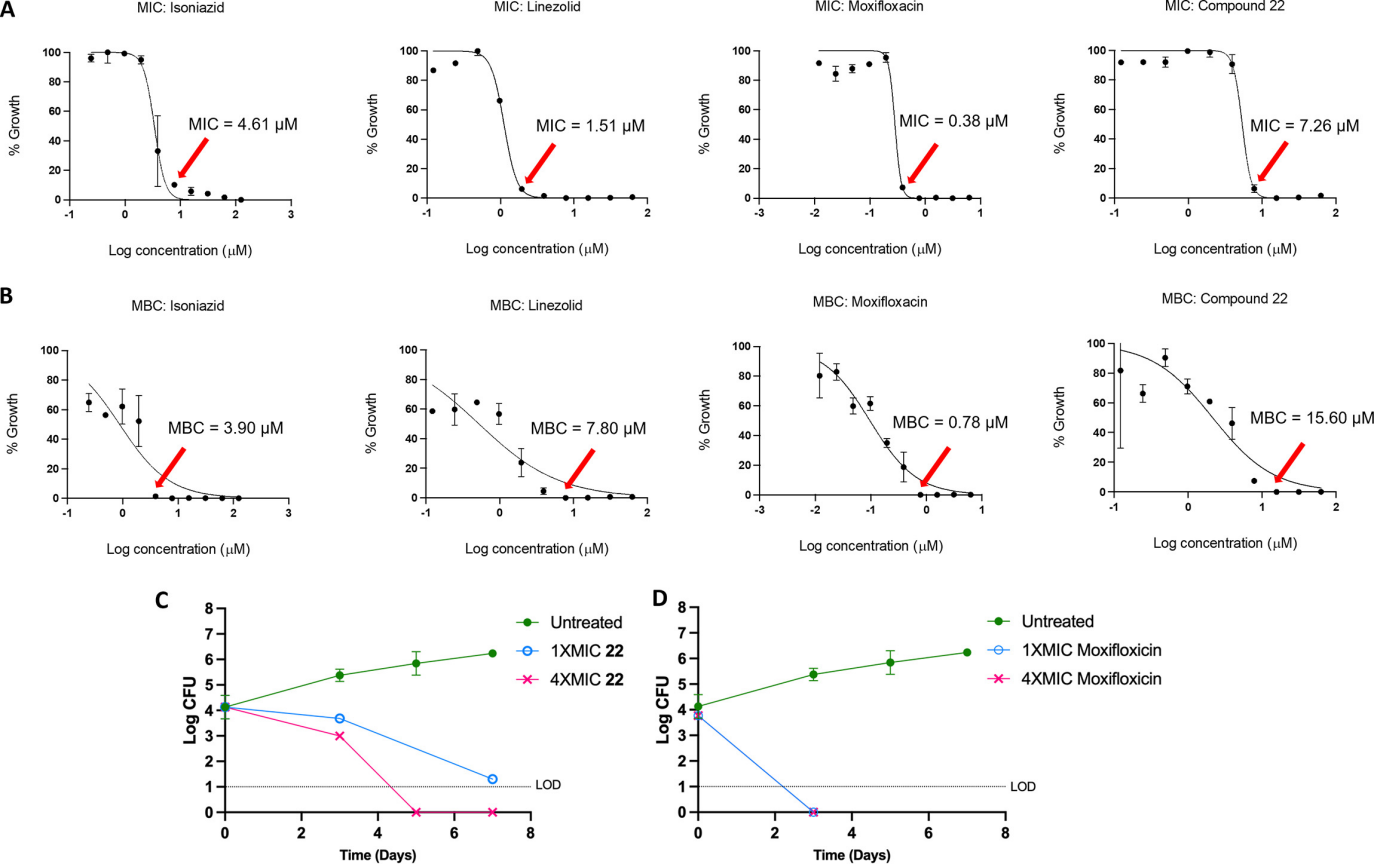
Cidality of compound 22. (A) Dose-response MICs for isoniazid, linezolid, and moxifloxacin and 22 against H37Rv over 7 days in Middlebrook 7H9 supplemented with OADC and glycerol. (B) MBC determinations for the same compounds. (C and D) Time- and concentration-dependent kill of replicating M. tuberculosis H37Rv by 22 (C) and moxifloxacin (D). The effect on viability was assessed by plating serial dilutions on standard Middlebrook 7H10 agar (supplemented with glycerol, OADC, and charcoal) at the indicated times followed by scoring CFU. Here, the MIC represents the liquid broth MIC. The results represent the means with standard deviations, and the limit of detection (LOD) is 10 bacilli.

Compound 22 showed a range of MIC values from 1.7 to 5.2 μM in the various M. tuberculosis growth medium compositions, the variation likely reflecting differential growth rates and perhaps variable influence of the DNA gyrase target. This activity is comparable to its activity against S. pneumoniae, S. aureus, and N. gonorrhoeae (MIC, 0.25, 0.5, and 2.0 μM, respectively) (11). To determine whether 22 exhibited bactericidal or bacteriostatic activity against M. tuberculosis H37Rv, the charcoal agar resazurin assay (CARA) was performed (22). Here, culture from a 7-day MIC broth assay is transferred to agar media containing activated charcoal to remove drug pressure. Cell growth is then measured after an additional 7-day incubation to determine the minimum bactericidal concentration (MBC). Positive controls for this assay consist of isoniazid, where the MBC is less than 4-fold the MIC, indicating bactericidal activity, and linezolid, where the MBC is greater than 4-fold the MIC, indicating bacteriostatic activity. The MBC for 22 was 2-fold higher than its MIC, a similar increase to what was observed for moxifloxacin, classifying it as a cidal compound (Fig. 1A and B; see also Fig. S1 in the supplemental material). Moreover, measurement of time-kill kinetics also revealed that 22 is cidal for M. tuberculosis in a time- and dose-dependent manner (3-log kill in 7 days at 1× MIC versus in 4 to 5 days at 4× MIC; Fig. 1C and D). Compound 22 did not show cross-resistance to the M. tuberculosis mutants containing canonical mutations associated with clinical resistance to INH, rifampicin, SQ109, moxifloxacin, inhibitors of MmpL3, and *d*-cycloserine (Table 4). In addition, it did not exhibit enhanced potency against a knockout of subunit 1 (Δ*cydA*) of cytochrome *bd* menaquinol oxidase or decreased sensitivity to a mutated *cytochrome bc*_1_ complex (QcrB A317T) in M. tuberculosis. Together, the latter two assays discount the possibility for disruption of mycobacterial respiration and *bc*_1_ menaquinol-cytochrome *c* oxidoreductase that are often seen as a mode of action in M. tuberculosis screening efforts and risk mammalian toxicity due to crossover inhibition of mitochondrial respiration (23, 24). Additionally, 22 showed intracellular activity against M. tuberculosis in murine macrophages with an IC_50_ (50% of the growth-inhibitory response for each readout) of 6.3 μM, less than 3-fold higher than the average *in vitro* broth MIC. The compound did not show toxicity up to the highest concentration tested of 100 μM against the macrophage cell line, in line with M. tuberculosis being affected rather than viability of the host.

**TABLE 4:** MIC_90_ values (μM) for M. tuberculosis drug-resistant strains^*a*^

Compound	H37Rv	MmpL3, G253E	MmpL3, G758A/G253E	ΔcydA	QcrB D317T	ATCC 3583 (INH resistance)	ATCC 35838 (Rif resistance)	ATCC 35826 (CS resistance)	DNA gyrase GyrA, A90V	DNA gyrase GyrA, G88C
22	1.0	2.0	2.0	2.0	3.9	3.9	2.0	2.0	0.98	0.98
SQ109 (MmpL3 control)	0.49	2.0	2.0							
NITDS9 (Δ*cydA* and QcrB control)	0.24			0.49	0.98					
Moxifloxacin (GyrA control)	0.24								7.8	0.49
Levofloxacin (GyrA control)	0.49								16	2.0
Rif (Rif and universal control)	0.002	0.001	0.001	0.0046	0.0046	0.006	>0.1	0.001	0.0023	0.0023
Isoniazid (INH control)	3.95					>62.5	7.8	3.4		

a INH, isoniazid; Rif, rifampicin; CS, cycloserine.

**FIG 2:**
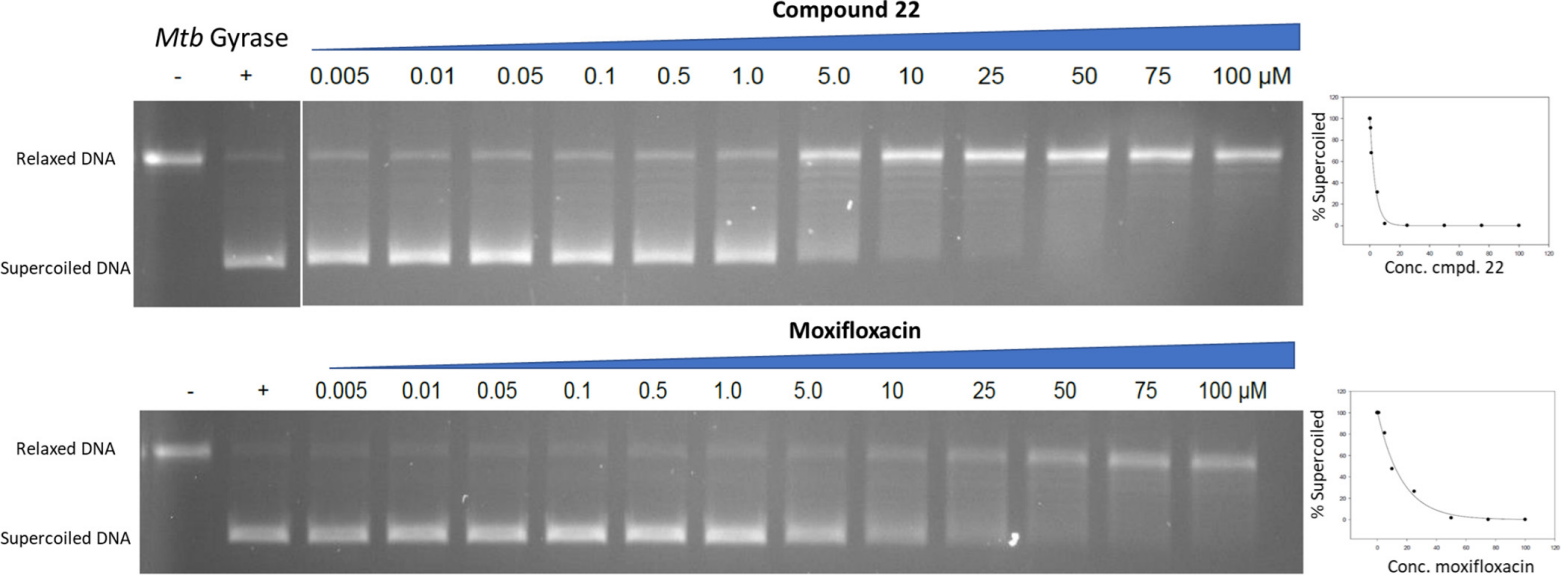
Effect of 22 and moxifloxacin on the conversion of relaxed DNA plasmid substrate to supercoiled DNA by purified M. tuberculosis (Mtb) DNA gyrase. Scanning by relative fluorescence of the bands affords the concentration-response curves to the right.

Next, 22 was assessed for inhibition of M. tuberculosis DNA gyrase supercoiling (25). 22 showed an IC_50_ of 2.0 μM (Fig. 2, average of two experiments), about 5-fold lower than the value for moxifloxacin (IC_50_, 10.5 μM), thereby supporting the presumed mode of action.

**FIG 3:**
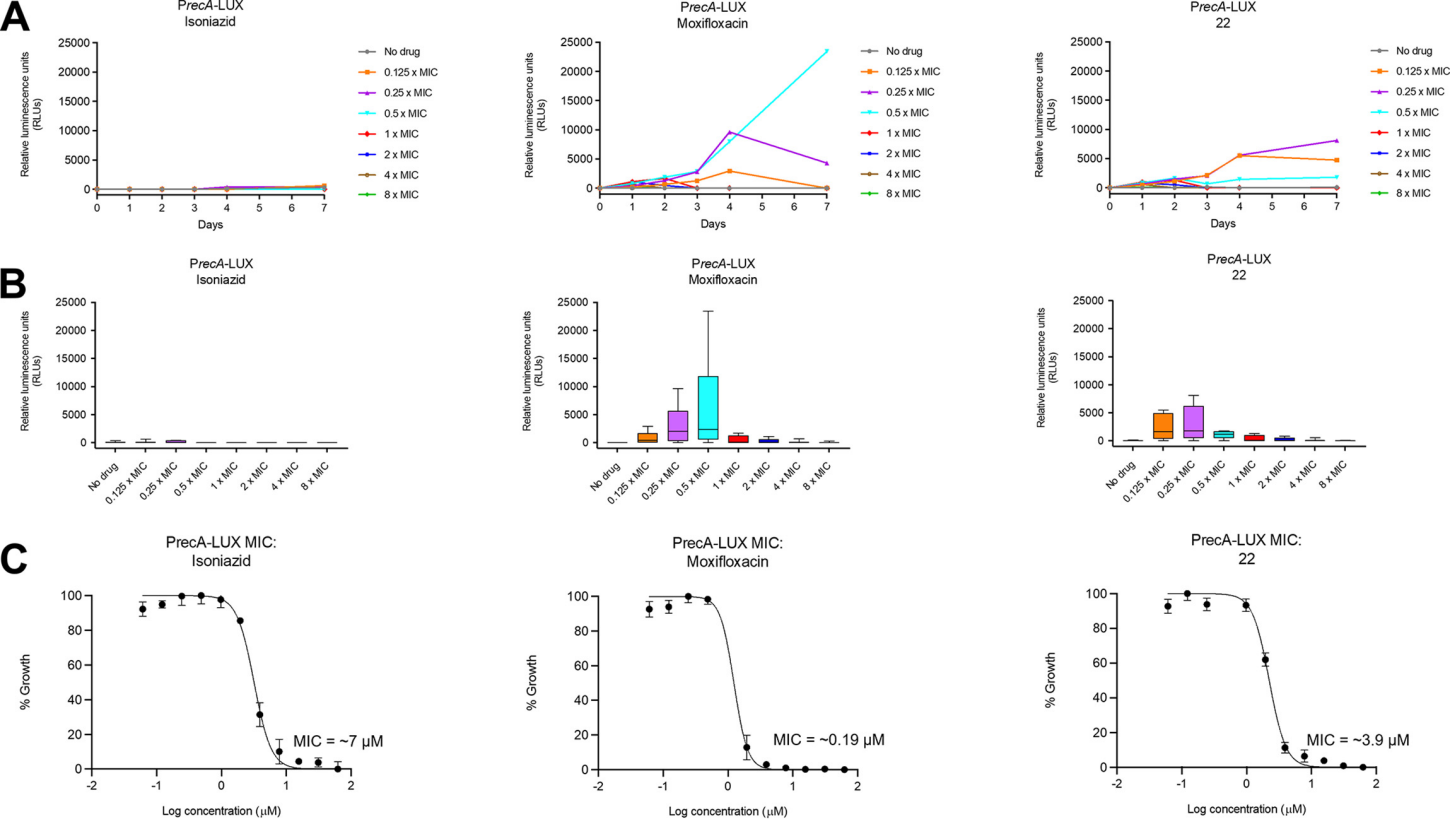
(A) Bioluminescence induced over 7 days by isoniazid, moxifloxacin, and 22 in H37Rv containing a modified *recA* promoter that drives the expression of DNA damage-inducible genes. (B) Total luminescence produced over 7 days for each concentration of isoniazid, moxifloxacin, and 22. (C) MIC determinations for isoniazid, moxifloxacin, and 22 against H37Rv (RecA).

To confirm that the lead compound 22 promotes DNA damage in M. tuberculosis cells *in vitro*, its activity was assessed in cells engineered with the *recA* promoter upstream of the bacterial luciferase *luxCDABE* operon from Photorhabdus luminescens (termed the P*recA*-LUX assay) (26). The *recA* gene serves to elicit the bacterial SOS response as a survival mechanism after sensing DNA damage; however, if the DNA damage is excessive, the SOS response accelerates cell death. As expected, 22 showed a strong bioluminescence signal (Fig. 3A and B) in the assay. A maximal effect was seen at 7 days with a concentration of 0.4 μM (4× lower than the MIC). Higher concentrations and more prolonged exposure to the drug led to cell death and a lower luminescent signal. The response in the P*recA*-LUX assay was qualitatively similar to that seen for moxifloxacin with perhaps a divergence at 0.5× MIC, where the signal was nearly 6-fold higher for moxifloxacin than 22. This divergence could reflect a higher cellular lethality for 22, limiting the *recA* response due to its higher target inhibitory potency. As a negative control, isoniazid, which inhibits cell wall biosynthesis, did not elicit significant luminescence in the assay as charted in Fig. 3, with the minimal signal seen likely due to general cell lethality (26). Conversely, the compounds were also assessed in a luminescence assay (termed the P*iniB*-LUX assay) designed to detect inhibitors of mycobacterial cell wall synthesis (Fig. S1) (26). Here, the *iniB* promoter of the *iniBAC* operon, which is upregulated in response to disruption of peptidoglycan, arabinogalactan, and mycolic biosynthesis, is cloned upstream of the *luxCDABE* operon (26). As expected, none of the DNA gyrase inhibitors, including 22, elicited a strong bioluminescence response in the P*iniB*-LUX assay compared to the strong signal observed for isoniazid.

### Physicochemical, pharmacokinetic, and safety profiles.

**FIG 4:**
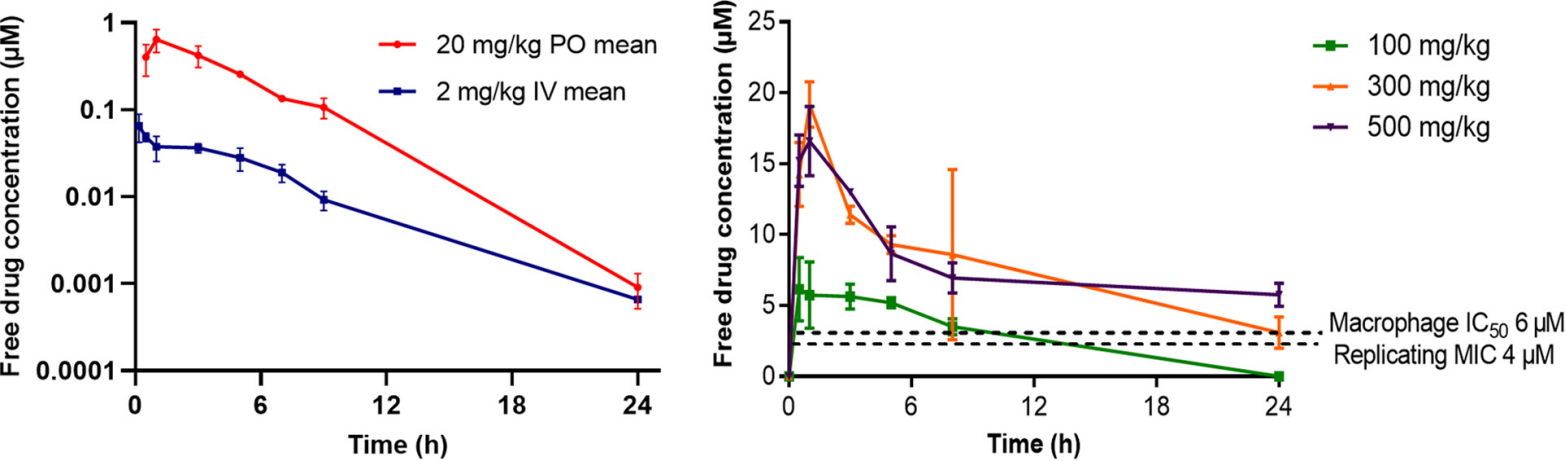
(Left) i.v. and p.o. PK curves in mice over 24 h on administration of 22 at 2 mg/kg and 20 mg/kg, respectively. (Right) PK of 22 on escalation of the p.o. dose. Dashed lines indicate the concentrations from *in vitro* data against M. tuberculosis H37Rv constitutively expressing DsRed in macrophages and against the replicating Erdman strain of M. tuberculosis.

Aiming to identify a compound to progress to *in vivo* efficacy studies, compounds were profiled for physicochemical properties, PK, and safety. All compounds showed favorable thermodynamic solubilities (at pH 6.5) ranging from 140 to 200 μM (the highest concentrated tested), in line with what would minimally be needed for an oral drug, the preferred mode of administration. The solubility of 22 itself was measured at 140 μM, and its PK parameters were determined in the mouse (Table 5 and Fig. 4). Intravenous (i.v.) dosing at 2 mg/kg of body weight gave a favorable blood clearance (CL_b_) of 14.3 mL/min/kg, with a mouse plasma protein binding (PPB) free fraction of 6.8%. This translates to an unbound clearance (CL_u_) of 210 mL/min/kg, 2- to 3-fold higher than that for moxifloxacin (90 mL/min/kg) (27). As a comparison, the human PPB free fraction for 22 was 15%, demonstrating species variability. Escalation of the oral (p.o.) dose showed greater than dose-proportional exposure (area under the concentration-time curve [AUC]) up to 300 mg/kg and saturation at the highest dose of 500 mg/kg, where exposure did not increase.

**TABLE 5:** PK properties of compound 22 in mouse^*e*^

Route of administration	Dose (mg/kg)	*C*_max_ (μg/mL)	*T*_max_ (h)	*t*_1/2_ (h)	CL_b_^*a*^ (mL/min/kg)	CL_u_^*b*^ (mL/min/kg)	*V*_ss_ (liters/kg)	AUC_0–∞_^*c*^ (h· μg/mL)	F^*d*^ (%)
i.v.	2			3.7	14.3	210	5.1	2.37	
p.o.	20	9.5	1.0					24.6	104
p.o.	100	47	2.8					343	
p.o.	300	133	1.0					1271	
p.o.	500	117	0.83					1435	

a Blood clearance.

b Unbound clearance based on PPB = 93.2%.

c Area under the curve.

d Oral bioavailibility.

e *T*_max_, time to maximum concentration of drug in serum; *t*_1/2_, half-life. *V*_ss_, volume at steady state.

### *In vivo* efficacy study.

**FIG 5:**
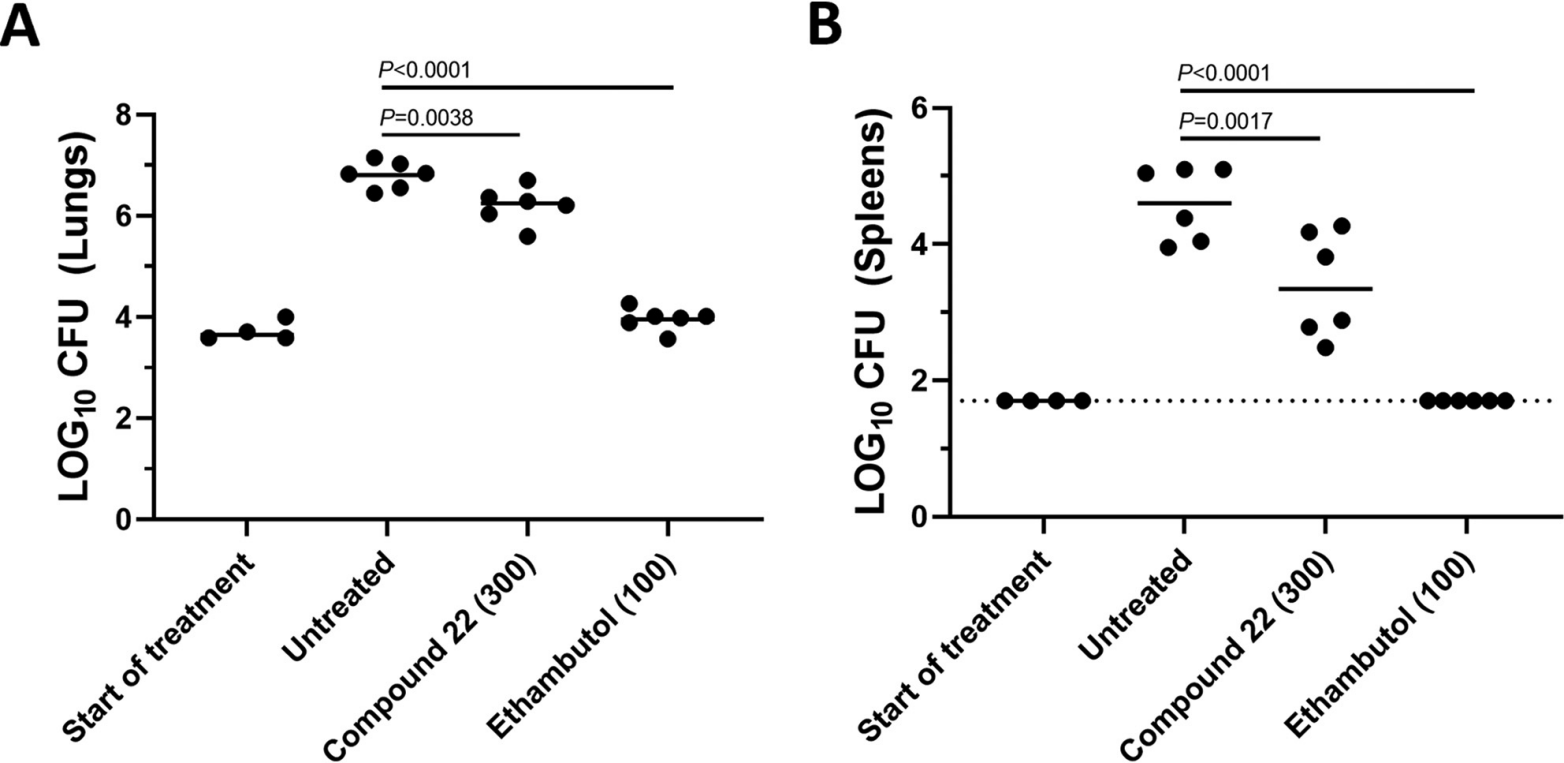
BALB/c mice were infected with M. tuberculosis Erdman Lux via a low-dose aerosol exposure. Treatment was started 7 days postaerosol and continued for 12 consecutive days. Drugs were administered once daily by oral gavage at the dose indicated. Plots represent log_10_ CFU determinations in lungs (A) and spleens (B) of individual BALB/c mice with the group means shown (solid horizontal line). The lower limit of detection in spleens was 1.7 log (indicated with a dashed line). *P* values determined by a one-way analysis of variance with a Dunnett’s multiple-comparison test are listed.

Due to good activity against M. tuberculosis and a favorable PK profile, 22 was evaluated for activity in a BALB/c acute TB murine efficacy model (28–30). In this model, mice are infected by low-dose aerosol, resulting in ∼2-log_10_ CFU M. tuberculosis Erdman pFCA-LuxAB (Erdman Lux, expressing luciferase) in the lungs of mice. The MIC against the Erdman strain was 4 μM (7H9 medium-ADC-glycerol) and 16 μM when the MIC was determined with the addition of 4% human serum albumin, indicating a modest shift due to PPB. At the start of treatment on day 7 postaerosol, the mean lung burdens had increased to 3.71 log_10_ CFU/lung (0.10 standard errors of the means [SEM]) with spleen burdens remaining below the lower limit of detection (Fig. 5). Mice were then dosed daily with 22 for 12 consecutive days by oral gavage in 0.2-mL volume at 300 mg/kg. Daily ethambutol at 100 mg/kg was included as a positive control owing to its established efficacy in this model (28). Untreated mice served as the negative control. Three days following the last day of oral dosing, the untreated negative controls and treated mice were sacrificed. The bacterial loads in lungs and spleens were determined following homogenization, serial dilution, and plating for CFU. In the untreated control mice, log_10_ lung and spleen burdens increased over time, as expected, to 6.81 ± 0.11 SEM and 4.50 ± 0.22 SEM, respectively. No mortalities occurred during the experiment, there were no problematic in-life observations, and mice generally gained weight, indicating the compound dosage was generally well tolerated. As expected, the positive-control ethambutol promoted a 2.6-log_10_ CFU decline (0.21 SEM) in lung burdens relative to the untreated controls. A much more modest, but statistically significant, 0.61-log_10_ decline in lung CFU burdens was seen for 22 (Fig. 5A), with similar results obtained based on the quantification of relative luminescence (RLU) from M. tuberculosis Erdman Lux in treated and untreated lung samples at the time of sacrifice (data not shown). In spleens, a larger overall decline in log_10_ CFU (i.e., Δ1.20 log_10_ CFU per spleen ± 0.21 SEM) was observed for 22, which was statistically significant (Fig. 5B). Blood was sampled on the ninth day of dosing at 1 and 24 h posttreatment for PK analysis, showing 22 concentrations (165 ± 58 μg/mL at the 1-h maximum concentration of drug in serum [*C*_max_]; Table S1) similar to what was seen for the same 300-mg/kg dose in healthy BALB/c mice. The drug concentration of 22 found in plasma at *C*_max_ exceeded the serum-shifted MIC of M. tuberculosis Erdman by ∼20-fold. However, based on projections of these data over PK curves from healthy BALB/c mice, the percent time above the MIC was likely only about 12 h. The free AUC/MIC (based on the untreated mouse PK) was 43. By comparison, moxifloxacin tested in an independent BALB/c acute TB efficacy study at 100 mg/kg (but not side by side with 22) reduced M. tuberculosis lung burdens to below the lower limits of detection in 3 of 6 mice (Table S2). The untreated control and the positive-control ethambutol (100 mg/kg) performed similarly to what is shown in Fig. 5, showing 6.50 ± 0.20 SEM and 4.15 ± 0.02 SEM log_10_ CFU, respectively, in the lung. Similarly, in a published study after 14 days of dosing, moxifloxacin (200 mg/kg) achieved a 3.3-log_10_ CFU reduction. PK parameters showed a free AUC/MIC of 85 and percent time above the MIC of about 17 h (31).

## DISCUSSION

Overall, the effects of the various media were minimal to the MIC values and suggest that binding to plasma proteins and DPPC (a major component of lung surfactant) would not have a large influence when compounds are introduced *in vivo*. The ideal compound for progression to a drug candidate would show high activity against the pathogen regardless of the media. The lack of cross-resistance for 22 (Table 4) with various mode-of-action M. tuberculosis agents is important for eventual stewardship to the clinic. It is gratifying that strains resistant to MmpL3 inhibitors, isoniazid, rifampicin, and cycloserine were fully susceptible to 22. As mentioned, there is also a preference to avoid compounds that target mycobacterial respiration with other compounds involved in various stages of clinical development including Q203 and cytochrome *bd* menaquinol oxidase (23, 24, 32).

Importantly, although antibacterial activity of both SPTs and FQs is due to stabilization of doubly cleaved DNA covalent complex of DNA gyrase at each of the cleavage sites, as demonstrated in other pathogens (33), their differential mode of inhibition is supported by the observation that bacterial strains resistant to FQs were fully susceptible to SPTs. This differential mode of inhibition has been attributed to the independence of SPTs to Mg^2+^ concentrations relative to what has been observed for FQs (33). Resistance of M. tuberculosis from clinical use of FQs most commonly occurs in DNA gyrase at Asp94, Ala90, Ser91, and Gly88, which are part of the quinolone resistance-determining region (QRDR) encompassed in codons 73 to 113 of GyrA (34–36). X-ray crystallography of an M. tuberculosis DNA gyrase/duplex DNA/moxifloxacin complex showed the inhibitor ketoacid chelates Mg^2+^ that otherwise bridges to Asp94 through water hydrates at each of the strand cleavage sites; hence, two molecules of moxifloxacin were seen (37). From the structure, GyrA A90V was believed to prevent the accommodation of the water and Mg^2+^, an interaction that therefore is not important for SPT binding. GyrA G88C was thought to alter the shape complementarity of FQ binding but must not play a role for SPT binding. The many other mutations leading to FQ resistance in the QRDR are not predicted to affect SPT binding, which would be worthwhile to assess using isogenic strains. X-ray crystallography of the S. aureus DNA gyrase isozyme with each of QPT-1, an SPT analogue, and moxifloxacin showed considerable overlap of the inhibitors, each positioned at the two cleavage sites (38). The tetrahydroquinoline scaffold of QPT-1 was intercalated between bases of the two DNA strands, as does the quinolone scaffold of moxifloxacin (although the stacking bases differed in the two structures), while the pyrimidinetrione moiety occupied a region not seen in the moxifloxacin structure. Despite this overlap of the scaffolds, there was no cross-resistance with the GyrA A90V and G88C mutant M. tuberculosis strains that lead to FQ resistance, similar to the situation described for SPTs and FQs for other pathogens. In N. gonorrhoeae and S. aureus, the susceptibility of FQ-resistant strains to SPTs was attributed to the absence of Mg^2+^ chelation or ligation for SPTs and thereby the lack of meaningful interactions within the QRDR, and it is likely that Mg^2+^ is not needed for SPT binding in M. tuberculosis as well. The inhibitory potency of 22 against the M. tuberculosis DNA gyrase was 5-fold higher than that seen for moxifloxacin, although antibacterial activity in culture was considerably (8-fold) lower. It is surmised that moxifloxacin has improved cell envelop permeability to reach the cytoplasm where the target is located. Alternatively, M. tuberculosis could metabolize SPTs, thereby mitigating activity. For future compound design, there would be value in characterizing SPT permeability relative to both cellular passive diffusion and active efflux as well as in investigating SPT metabolism in M. tuberculosis culture. The action of both SPTs and FQs against nonmycobacterial species involves an interplay where the two topoisomerase targets, DNA gyrase and Topo-IV, are inhibited, albeit to various degrees. As M. tuberculosis DNA gyrase carries out both DNA supercoiling and decatenation, the primary action of (nonmycobacterial) DNA gyrase and Topo-IV, the potential for mitigating resistance due to dual targeting is unfortunately removed.

It follows that 22 would elicit the bacterial SOS response mediated by recognition of DNA damage by the *recA* promoter, as seen for moxifloxacin (39). Across bacterial species, the SOS response contributes to cell lethality as a reaction to DNA-damaging agents, including to FQs. This response has also been linked with faster killing kinetics and bactericidal (versus bacteriostatic) activity (31). Other DNA gyrase inhibitors, including ATP competitive inhibitors such as novobiocin, show slow killing kinetics in other (nonmycobacterial) bacteria (33, 40, 41). Compound 22 showed an MBC only 2-fold higher than its MIC. Under replicating conditions, a ≥4-fold increase of a compound’s MBC compared to the MIC indicates bacteriostatic activity and is called a static window (22). Additionally, 22 showed cidality *in vitro* at 1 to 8× the MIC, where the log CFU ranged from 1 to 3 logs lower relative to the initial inoculum, similar to what was observed for moxifloxacin. Cidality was also observed for other SPTs, including zoliflodacin, against S. aureus and N. gonorrhoeae, in line with what would be expected for antibacterials eliciting the SOS response (12, 18). It is envisioned that clinical performance against M. tuberculosis would be enhanced with a cidal compound, which might also play a role in eliminating the pathogen from when M. tuberculosis is in the nonreplicating state. That 22 showed intracellular activity in macrophages further bodes well for the series.

The structure-activity relationship (SAR) for SPT analogues against M. tuberculosis diverged from those published against other pathogens, including against N. gonorrhoeae, the target of zoliflodacin. Zoliflodacin showed a MIC against N. gonorrhoeae that was 8-fold lower than that for 22, yet the latter was about 6-fold more active against M. tuberculosis. The MIC values for zoliflodacin were similar to those for 5 against N. gonorrhoeae and a series of Gram-positive pathogens (S. aureus, S. pneumoniae, and S. pyogenes), yet 5 was consistently about 2-fold more active across the MIC assays carried out against M. tuberculosis. Compound 5 notably has an oxazolidinone ring as the R substituent, as does zoliflodacin, but it differs by the positioning of a methyl group. Zoliflodacin also showed comparable activity against S. aureus, S. pneumoniae, and S. pyogenes to the triazole compound 14 (18), but here it was 16-fold less active against M. tuberculosis (Table 2). That the SAR diverges among pathogens offers a strong rationale to optimize for M. tuberculosis activity in analogue programs. What is not clear is whether this divergence is due to target potency or permeability, that is, the ability of a compound to transverse the bacterial cell membranes to reach the cytoplasm.

Using a BALB/c acute TB murine infection model, 22 was found to promote a small but statistically significant reduction of M. tuberculosis burden in lung, just beyond standard deviation, relative to the untreated control. Activity was considerably lower than what has been seen for moxifloxacin. PK in healthy mice predicted a free drug concentration of about 2-fold over the MIC for over 8 h, which may not be sufficient for demonstration of better efficacy. As the PK/PD driver of SPTs against M. tuberculosis is not known, there is a better correlation for zoliflodacin for free AUC/MIC than percent time above the MIC for S. aureus mouse models of skin infection (11). The *C*_max_ in the diseased mice was quite variable but in line with what was seen in healthy mice. There was minimal or no shift in MIC values in the presence of DPPC, the principle (∼80%) component of lung surfactant, which might have accounted for decreased susceptibility if there was a tight interaction (14). The levels of compound in the lung epithelial lining fluid or homogenate were not measured, which could also be lower than what was determined in the blood during PK measurements. Compound 22 also showed a higher level of activity in macrophages that is known to be the main harbor for the pathogen in the mouse acute model (13, 42). There was a more discernible effect seen for 22 in the bacterial load of the spleen during the efficacy experiment, demonstrating an *in vivo* proof of concept.

### Conclusions.

Clearly, more analogue optimization work is necessary around the SPT class of DNA gyrase inhibitors to identify compounds that would have value in treating TB in humans. Considerable headway toward this would be seen if higher M. tuberculosis activity could be achieved, and work toward this end will be duly reported. Being able to assess a small collection of SPTs synthesized in programs directed against Gram-positive and fastidious Gram-negative pathogens against M. tuberculosis has nonetheless been able to drive consideration into repurposing the series. However, the SAR for M. tuberculosis differs significantly enough that a directed analogue optimization program is necessary. Notably, 22 demonstrated inhibition of M. tuberculosis DNA gyrase and was a more potent inhibitor of M. tuberculosis DNA gyrase than moxifloxacin, although its whole-cell activity was considerably lower. This emphasizes that drug design efforts need to optimize bacterial permeability as much as target potency toward deriving lower MIC values (43). DNA damage was indicated for 22 by the *PrecA*-Lux assay, consistent with the DNA gyrase mode of action. Ultimately, generating spontaneous resistant mutants to 22 would be called for to support further the DNA gyrase mode of action, work that will be reported in due course with a more active SPT analog. That SPTs are not cross-resistant to FQs or other classes of TB drugs positions the series as one that can battle problematic issues of resistance. Inhibiting DNA gyrase differently than FQs without cross-resistance is tantamount to having a different mode of action. Work is in progress to characterize the SPT binding mode to DNA gyrase of M. tuberculosis with the expectation that it involves stabilization of the doubly cleaved DNA complex independent of Mg^2+^, as demonstrated for other bacterial pathogens (33). The recent clinical success shortening the treatment duration for TB with moxifloxacin also underscores the importance of expanded research investigations into DNA gyrase inhibiting anti-M. tuberculosis agents. Having zoliflodacin, the most advanced member of the SPT class now in phase 3 trials, suggests that it is possible to integrate favorable PK and safety attributes necessary for ultimate success in the clinic. Hence, it can be anticipated that further considerations will be given to the SPT class on the road to finding new and valuable treatments for TB.

## MATERIALS AND METHODS

### Determination of MICs.

MICs were determined in six Middlebrook 7H9-based media: glucose-albumin dextrose catalase (ADC)-Tween 80, glucose-casitone-tyloxapol, glucose-BSA-tyloxapol, DPPC-cholesterol-tyloxapol, and cholesterol-BSA-tyloxapol. The compounds were serially diluted in the desired medium 2-fold across wells in a 96-well clear round-bottom plate, in duplicate. The M. tuberculosis cultures were grown to an optical density at 600 nm (OD_600_) of 0.5 in the required medium and diluted 10^3^-fold in the same medium to get a starting inoculum of ∼10^5^ to 10^6^ cells. Next, 50 μL was added to each well, approximating 10^5^ cells per well. Isoniazid, rifampin, and dimethyl sulfoxide (DMSO) (drug free) were used as positive/negative controls. The plates were incubated for a total of 7 days at 37°C in Ziploc bags. At day 7, cell growth was estimated using an inverted enlarging mirror plate reader with the MIC taken as the lowest compound concentration that inhibited all visible growth. MIC values against M. tuberculosis Erdman were determined as described above with and without added 4% (wt/vol) human serum albumin in Middlebrook 7H9-based medium supplemented with 0.2% (vol/vol) glycerol, 0.5% (wt/vol) BSA, 0.2% (wt/vol) glucose, 0.0003% (wt/vol) catalase using an alamarBlue endpoint (MABA) readout.

### CARA and time-kill kinetics.

A dose-response MIC against M. tuberculosis H37Rv was set up in duplicate as described above using Middlebrook 7H9 medium supplemented with oleic acid-albumin-dextrose-catalase (OADC) and glycerol with the M. tuberculosis culture diluted 100-fold in the same medium. Positive and negative controls for this assay consisted of isoniazid (bactericidal control), linezolid (bacteriostatic control), and DMSO (drug-free control). After a 7-day incubation, 10 μL from each well of the MIC plates was transferred to 96-well plates containing Middlebrook 7H10 agar supplemented with OADC, glycerol, and charcoal. Plates were then incubated for 7 days at 37°C, followed by the addition of 50 μL resazurin in each well for 1 h. Plates were scored using the SpectraMax i3x (Molecular devices) with the average fluorescence extrapolated using Softmax Pro (version 6.5.1) in each well. The observed fluorescence in the assay was adjusted for background fluorescence, recorded using rifampicin at 0.15 μM, where there is 100% inhibition of bacterial growth. The activity of 22 on M. tuberculosis was also tested *in vitro* to score the bactericidal or bacteriostatic nature. M. tuberculosis H37Rv cultures growing in Middlebrook 7H10-glycerol-ADC were exposed to various concentrations of compounds. At indicated time points, the samples were collected and washed, and appropriate dilutions were plated on Middlebrook 7H10-glycerol-OADC-charcoal (0.4%, wt/vol) to score the viable bacteria per published protocols (22). The results are shown as mean values and standard deviations from three replicates, and the limit of detection is 10 bacilli.

### Bioluminescence assay.

The M. tuberculosis strain carrying the *PrecA*-LUX (genotoxicity reporter) construct was grown at 37°C in standard Middlebrook 7H9 medium supplemented with glucose-ADC (Difco), 0.05% Tween 80, and 20 μg/mL kanamycin (KAN). The culture was grown to an optical density at 600 nm of ±0.4 and was diluted 1:10 prior to inoculation. The assay was performed using a modified version of the standard broth microdilution method. Twofold serial dilutions of the test material and the assay control drugs were prepared in a flat-bottomed 96-well opaque microtiter plate, in a volume of 50 μl, and 50 μL of the relevant diluted M. tuberculosis reporter culture was added to each well in the plate (including the assay control wells) for a final volume of 100 μl per well. The assay plate was incubated at 37°C with 5% CO_2_ and humidification for 7 days. Each plate contained a standard set of controls: growth medium with M. tuberculosis cells (to determine the minimum background luminescence), growth medium only, moxifloxacin at 10 concentrations, ranging from 1.25 to 0.003 μg/mL, and isoniazid at 1 to 0.002 μg/mL. The number of relative luminescence units (RLU) was determined at specific time intervals (every 24 h) over a range of drug concentrations spanning the known MIC_90_ using a SpectraMax i3x plate reader (Molecular Devices Corporation).

### M. tuberculosis DNA gyrase supercoiling assay.

One unit (the amount of enzyme required to fully supercoil the substrate) of M. tuberculosis gyrase was incubated with 0.5 μg of relaxed pBR322 DNA in a 30-μL reaction mixture at 37°C for 30 min under the following conditions: 50 mM HEPES, KOH (pH 7.9), 6 mM magnesium acetate (MgOAc), 4 mM dithiothreitol (DTT), 1 mM ATP, 100 mM potassium glutamate, 2 mM spermidine, and 0.05 mg/mL BSA. Each reaction was stopped by the addition of 30 μL chloroform–iso-amyl alcohol (24:1) and 20 μL stop dye (40% sucrose, 100 mM Tris-HCl [pH 7.5], 10 mM EDTA, 0.5 μg/mL bromophenol blue) before being loaded on a 1.0% TAE (0.04 mM Tris-acetate, 0.002 mM EDTA) gel run at 80 V for 3 h. Bands were visualized by ethidium staining for 10 min, destained for 10 min in water, analyzed by gel documentation equipment (Syngene, Cambridge, UK), and quantitated using Syngene GeneTools software. Raw gel data (fluorescent band volumes) collected from Syngene GeneTools gel analysis software were calculated as a percentage of the 100% control (the fully supercoiled DNA band) and converted to percent inhibition. The raw gel data were analyzed using SigmaPlot version 13 (2015). The global curve fit nonlinear regression tool was used to calculate IC_50_ data using the following equation: exponential decay, single, two-parameter *f* =* a* × exp(−*b* × *x*).

### M. tuberculosis macrophage assay.

The macrophage assay was carried out as described previously (44). Briefly, murine RAW 264.7 cells were infected with M. tuberculosis expressing DsRed at a multiplicity of infection of ∼1. Infected cells were exposed to compounds for 72 h and imaged at ×4 magnification using an ImageXpress Micro high-content screening system (Molecular Devices). Bacterial growth was measured by fluorescence using a Texas red filter. Macrophage viability was monitored by staining nuclei by SYBR green I using a fluorescein isothiocyanate (FITC) filter. The integrated intensity of each channel was determined using MetaXpress software and used to calculate growth/viability with respect to control wells. Curves were fitted using the Levenberg-Marquardt algorithm. The IC_50_ was defined as the compound concentration that resulted in 50% of the growth-inhibitory response for each readout.

### Mouse PK.

All protocols for PK studies used male CD57/B6 mice, were conducted by following guidelines and policies as stipulated in the UCT Research Ethics Code for Use of Animals in Research and Teaching, after review and approval of the experimental protocol by the UCT Senate Animal Ethics Committee (protocol FHS-AEC 013/032), and were carried out at the Research Animal Facility located at the Division of Clinical Pharmacology, University of Cape Town, Cape Town, South Africa. Compound 22 (2.0 mg/kg) was administered intravenously to 3 mice as a bolus of dimethylacetamide (DMA)-polyethylene glycol-dextrose (in 5% water) (D5W): 15/50/35. Oral doses (20, 100, 300, and 500 mg/kg) were each administered to 3 mice as an aqueous suspension containing 0.5% (wt/vol) carboxymethylcellulose (CMC) and 0.2% (vol/vol) Tween 80 in water. Mice were not fasted overnight. Animals were permitted access *ad libitum* to water. Blood samples were collected from the mice into heparinized microcentrifugation tubes at 0.17 (i.v. only), 0.5, 1, 3, 5, 7, 9, 12, and 24 h after dosing and stored frozen (−80°C) until analyses. Frozen mouse whole-blood samples were thawed, and 30 μL was extracted by protein precipitation using 200 μL of acetonitrile containing 10 ng/mL internal standard, vortexed, and centrifuged. Calibration standards and quality controls were extracted by following the same procedure. Supernatants were injected onto the column for LC-MS/MS analysis. The compound concentration was determined using an LC-MS/MS (AB Sciex API3200 triple quadrupole mass spectrometer coupled to an Agilent 1100 series high-performance liquid chromatograph). Chromatographic separation was conducted with a Phenomenex Hydro-RP column (2.5-μm particle size; 50- by 2.1-mm internal diameter), maintained at 30°C. The mobile phase consisted of 0.1% (vol/vol) formic acid in water and 0.1% (vol/vol) formic acid in acetonitrile mixed using a linear gradient. Sample concentrations were calculated by comparison to calibration standards prepared and assayed under the same conditions. Elution of analytes was confirmed by multiple reaction monitoring in positive electrospray ionization mode. The accuracy, precision, and recovery for each compound were within acceptable limits. Pharmacokinetic parameters were calculated by noncompartmental analysis using PK Solutions 2.0 (Summit Research Services, Montrose, CO, USA) using a method based on curve stripping. Mouse plasma protein binding was determined by equilibrium dialysis from a 10 μM solution of 22 in a Dianorm plasma well incubated at 37°C for 16 h. Free fractions were calculated from ratios of drug concentration in buffer and plasma wells determined by LC-MS/MS. The fraction unbound (f_u_) = peak area ratio (buffer sample/internal standard [IS])/peak area ratio (plasma sample/IS).

### Murine model of acute TB infection.

All manipulations of mice in the TB disease model were performed at Colorado State University in a certified animal biosafety level 3 facility, in accordance with guidelines of the Colorado State University Institutional Animal Care and Use Committee. Ethics oversight was provided by the Colorado State University Animal Care and Use Program, which is PHS assured (A3572-01), USDA registered (84-R-0003), and AAALAC accredited (number 000834). The IACUC approved CSU protocol number is 18-7912A. Six- to 8-week-old female specific-pathogen-free BALB/c mice were purchased from Jackson Laboratories (Bara Harbor, ME). Mice were infected with M. tuberculosis Erdman pFCA-LuxAB (Erdman Lux) via a low-dose aerosol exposure in a Glas-Col aerosol generation device (Glas-Col Inc., Terre Haute, IN). Each treatment group consisted of six mice. Compound 22 was formulated in 0.5% carboxymethylcellulose (Sigma) with 0.2% Tween 80 (Fisher Scientific). Ethambutol was prepared in sterile water. Treatment was started 7 days postaerosol and continued for 12 consecutive days. Drugs were administered by oral gavage in a 0.2-mL volume at 300 mg/kg and 100 mg/kg for 22 and ethambutol, respectively. For endpoint analysis, mice were euthanized 3 days following the end of treatment, and lungs and spleens were collected (last drug dose on a Friday and mouse tissues aseptically harvested on Monday). The left lung lobe (1/3 of the lung by weight) was homogenized for enumeration of CFU by plating dilutions of the organ homogenates on Middlebrook 7H11 medium supplemented 10% (vol/vol) OADC, 0.01 mg/liter cycloheximide, and 0.05 mg/liter carbenicillin, as was a portion of the spleen tissue. The data were expressed as mean log_10_ CFU ± standard errors of the means (SEM) for each group. Statistical analysis was by one analysis of variance with Dunnett’s posttest to control for multiple comparisons (SigmaPlot, San Jose, CA). Values were considered significant at the 95% confidence level.

In a separate experiment (see Table S2 in the supplemental material), treatment with ethambutol or moxifloxacin was started 7 days postaerosol and continued for 12 consecutive days. Ethambutol and moxifloxacin were prepared in sterile water. Drugs were administered by oral gavage in a 0.2-mL volume at 100 mg/kg for either ethambutol or moxifloxacin.
