# Supplementary material for: Spiropyrimidinetriones: a Class of DNA Gyrase Inhibitors with Activity against Mycobacterium tuberculosis and without Cross-Resistance to Fluoroquinolones
Source: Antimicrob Agents Chemother. 2022 Mar 10;66(4):e02192-21. doi: 10.1128/aac.02192-21 (PMC9017349; doi:10.1128/aac.02192-21)
Supplement: SUPPLEMENTAL FILE 1 — Fig. S1 and Tables S1 and S2. Download aac.02192-21-s0001.pdf, PDF file, 0.3 MB [file aac.02192-21-s0001.pdf]

Supplemental Material:

Spiropyrimidinetriones: a class of DNA gyrase inhibitors with activity against Mycobacterium tuberculosis and without cross-resistance to fluoroquinolones

Gregory S. Basarab[a,b], Sandeep Ghorpade[b], Liezl Gibhard[a], Rudolf Mueller[b], Mathew Njoroge[a], Nashied Peton[b], Preshend Govender[b], Lisa M Massoudi[c], Gregory Thomas Robertson[c], Anne J. Lenaerts[c], Helena Ingrid Boshoff[d], Douglas Joerss[e], Tanya Parish[e], Thomas F. Durand-Reville[f], Manos Perros[f], Vinayak Singh[b,g], Kelly Chibale[b,g]

Affiliations:

[a]Drug Discovery and Development Centre (H3D), Old Main Building Groote Schuur Hospital, University of Cape Town, Observatory, Cape Town, 7935, South Africa

[b]Drug Discovery and Development Centre (H3D), Department of Chemistry, University of Cape Town, Rondebosch 7701, South Africa

[c]Mycobacteria Research Laboratories, Department of Microbiology, Immunology and Pathology, 200 West Lake Street, Colorado State University, Fort Collins, CO 80523-1682, USA

[d]Tuberculosis Research Section, National Institute of Allergy and Infectious Diseases, Bethesda, Maryland 20892, USA

[e]Infectious Disease Research Institute, 1616 Eastlake Ave E, Seattle, WA 98102, USA and Center for Global Infectious Disease, Seattle Children's Research Institute, 307 Westlake Ave N, Seattle, 90810, USA

[f]Entasis Therapeutics, Inc., 35 Gatehouse Drive, Waltham, Massachusetts 02451, USA

[g]South African Medical Research Council Drug Discovery and Development Research Unit, Department of Chemistry and Institute of Infectious Disease and Molecular Medicine, University of Cape Town, Rondebosch 7701, South Africa

Corresponding Authors: Address correspondence to Gregory S. Basarab (greg.basarab@uct.ac.za) and Kelly Chibale (kelly.chibale@uct.ac.za)

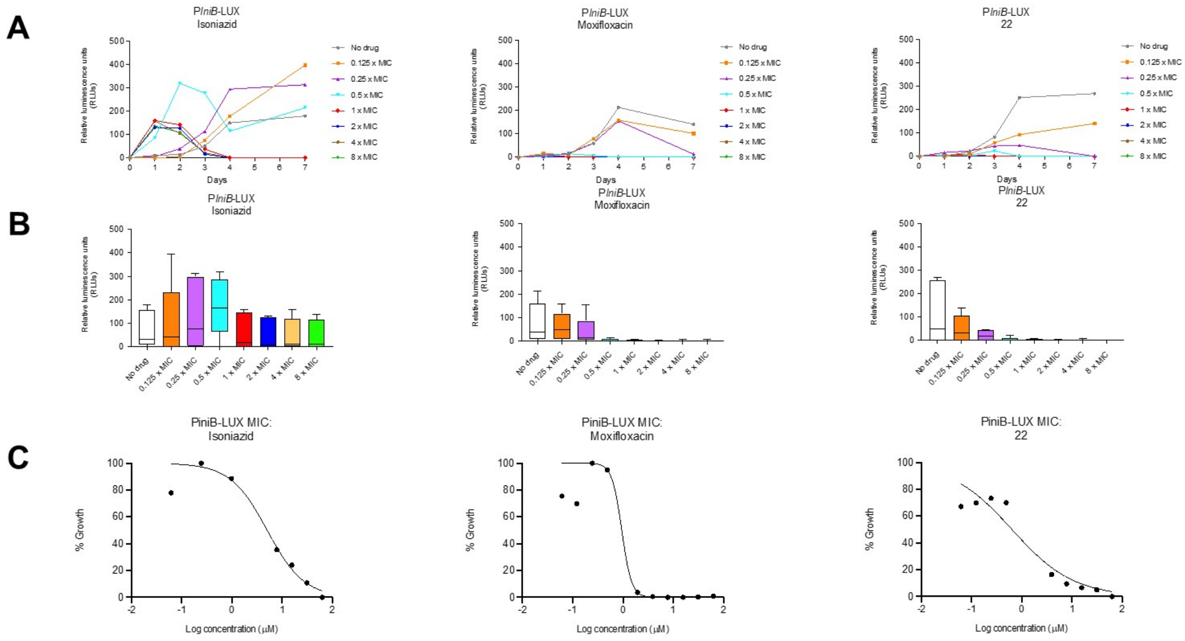

Figure S1: Compound **22** does not affect *Mycobacterium tuberculosis* (*Mtb*) cell wall biosynthesis. (A) Luminescence produced by H37Rv containing a modified IniB promoter (that drives the expression of cell wall damage-inducible genes) over 7 days of Isoniazid, Moxifloxacin and **22**. (B) Luminescence range produced (over 7 days) for each concentration of Isoniazid, Moxifloxacin and **22**. (C) MIC determination for Moxifloxacin, Isoniazid **22** against H37Rv (IniB) over 7 days.

Table S1: Concentrations of **22** in plasma collected from *Mtb* infected BALB/c mice.

Treatment	Mouse	Time point (hr post dose)	Found Concentration (ng/mL)	Average Conc. Found (ng/mL)
Group 2	A	1	229000	
	B	1	117000	165000
Compound **22**	C	1	148000	
300 mg/kg QD	D	24	879	
	E	24	994	6100
	F	24	16300	

Table S2: Moxifloxacin and ethambutol in the acute mouse model of *Mtb* infection.

Group	Dose (mg/kg)	Days[a]	Bacterial burden in lung (log_{10} CFU ± SEM)	*n*[b]
Infection control		1	2.86 ± 0.03	3/3
Start of treatment		7	4.16 ± 0.04	5/5
Untreated		21	6.51 ± 0.20	6/6
Moxifloxacin	100	21	< 1.67 ± 0.06	3/6
Ethambutol	100	21	4.15 ± 0.02	5/5

[a] Days post aerosol infection

[b] *n*, number of mice showing viable bacteria above the lower limit of detection (1.57 log_{10} CFU) at time of sacrifice/total number of animals tested
